# Revealing deep evolutionary relationships between RNA viruses using predicted structural models of viral RNA polymerases

**DOI:** 10.1093/molbev/msag088

**Published:** 2026-04-07

**Authors:** Heli A M Mönttinen, Janne J Ravantti, Richard Mayne, Peter Simmonds, Minna M Poranen

**Affiliations:** Institute of Biotechnology, Helsinki Institute of Life Science, University of Helsinki, Helsinki FI 00014, Finland; Department of Molecular and Integrative Biosciences, Faculty of Biological and Environmental Sciences, University of Helsinki, Helsinki FI 00014, Finland; Nuffield Department of Medicine, University of Oxford, Oxford, UK; Institute of Biomedicine, University of Turku, Turku FI 20014, Finland; MRC-University of Glasgow Centre for Virus Research, University of Glasgow, Glasgow, UK; Department of Molecular and Integrative Biosciences, Faculty of Biological and Environmental Sciences, University of Helsinki, Helsinki FI 00014, Finland

**Keywords:** RNA-dependent RNA polymerase, virus evolution, structure-based phylogeny, AlphaFold

## Abstract

The RNA-dependent RNA polymerase (RdRP) is the only homologous gene shared among current members of the kingdom *Orthornavirae* in the realm *Riboviria.* It is therefore used as a hallmark gene to infer their evolutionary relationships and to guide their taxonomic classification. While sequence similarity between RNA viruses is often limited and sequences problematic to align, the conservation between the three-dimensional tertiary structures of viral RdRPs is notable, supporting analysis of deep evolutionary relationships. Nevertheless, the limited availability of experimental RdRP structures restricts structure-based phylogenetic analyses. We used the protein structure prediction algorithm AlphaFold to alleviate this restriction and predicted structure models for 989 viral RdRPs. Through structural alignment with Homologous Structure Finder, we identified 211 structurally equivalent residues for RdRPs, representing 96 virus genera recognized by the International Committee on Taxonomy of Viruses. These equivalent residues were used to deduce a comprehensive structure-based phylogenetic tree for viral RdRPs, which was validated using a jackknifing approach developed in this study. For comparison, structural phylogenies were inferred using alignments produced with FoldTree and FoldMason software. The resulting trees mostly support the current taxonomic assignments of RNA viruses at the class rank. However, they do not support the monophyly of phyla *Pisuviricota* and *Duplornaviricota.* Furthermore, flaviviruses frequently group apart from other members of *Kitrinoviricota*. The conservation of protein structures over long periods of evolutionary time, when detectable sequence homology may be lost and sequence alignment problematic, supports the use of protein structure comparison methods for demonstrating the deeper evolutionary histories of RNA viruses.

## Introduction

RNA-dependent RNA polymerases (RdRPs) are essential enzymes responsible for replicating and transcribing viral RNA genomes. Despite the vast diversity of RNA viruses, all structurally characterized viral RdRPs share a conserved right-hand-shaped architecture comprising palm, fingers, and thumb subdomains (Hansen et al. 1997; Tao et al. 2002; Pflug et al. 2014), and have a shared evolutionary origin that is distinct from cellular polymerases. Consequently, it has been proposed that the RdRP might be used as a hallmark gene for the taxonomic classification of viruses within the kingdom *Orthornavirae* by the International Committee on Taxonomy of Viruses (ICTV) (Wolf et al. 2018; Neri et al. 2022).

Early sequence-based comparisons of RdRP genes identified seven conserved sequence motifs (A–G) (Poch et al. 1989; Bruenn 2003; Gorbalenya 2018), which were later expanded into broader structural homomorphs (Lang et al. 2013). These homomorphs correspond to conserved protein folds within the palm and fingers subdomains, which are critical for the correct orientation of incoming nucleotides, template binding, and catalysis (Butcher et al. 2001; Ng et al. 2008; Lang et al. 2013; Te Velthuis 2014). The catalytic aspartates in motifs A and C within the palm subdomain (Poch et al. 1989; Ng et al. 2008; Te Velthuis 2014) are the only strictly conserved amino acids among RdRPs; the remaining sequence motifs exhibit high variability, making the alignment and inference of a comprehensive, sequence-based phylogenetic tree for viral RdRPs highly challenging and potentially arbitrary (Holmes and Duchêne 2019).

The higher-order taxonomy of RNA and retrotranscribing viruses in the realm *Riboviria* was initially developed based on a sequence-based phylogenetic tree that divided RdRPs into five major groups (Wolf et al. 2018). This tree was inferred from 4,640 RdRP and 1,028 reverse transcriptase sequences using a stepwise method, starting with the creation of profiles for subclusters of sequences. These profiles were used to build a preliminary guide-tree through profile–profile comparisons. Subtrees of this guide tree were then used to guide the HHalign procedure. For each cluster with three or more sequences, a maximum-likelihood phylogenetic tree was inferred with WAG substitution matrix and gamma-distributed rates (Wolf et al. 2018). Finally, the global tree was inferred for representative sequences from each cluster using the maximum likelihood method with the LG substitution matrix and gamma-distributed site rates (Wolf et al. 2018). This tree delineated five major branches of RNA viruses and guided the higher-order taxonomic classification of RNA viruses into five phyla and the establishment of 14 new classes under these phyla *Lenarviricota* (4 classes), *Duplornaviricota* (3 classes), *Negarnaviricota* (6 pre-existing classes), *Kitrinoviricota* (4 classes), and *Pisuviricota* (3 classes). The main branches (phyla) were monophyletic with high bootstrap support value (>0.7) apart from *Duplornaviricota* that was paraphyletic with the *Negarnaviricota* (>0.7 support) (Wolf et al. 2018). However, concerns have been raised about the robustness of the RdRP sequence alignment, particularly regarding its ability to resolve the early evolution of RNA virus (Holmes and Duchêne 2019). Nevertheless, the current taxonomy of RNA viruses (kingdom *Orthornavirae*) at higher taxonomic ranks is largely based on this single sequence analysis of viral RdRPs.

A similar approach was later applied to a much larger dataset of RdRP sequences predicted from metatranscriptome data (77,520 sequences) (Neri et al. 2022), which mostly supported the monophyly of the five major phyla. However, several viral families (*Cystoviridae*, *Picobirnaviridae*, *Reoviridae*, *Flaviviridae*, and *Barnaviridae*) frequently violated the monophyly of the previously established phyla. For example, *Duplornaviricota* was only monophyletic when RdRP sequences of *Cystoviridae*, which frequently clustered in this analysis with members of the class *Duplopiviricetes* (phylum *Pisuviricota*), were excluded. The study also suggested a number of additional high rank taxa for RNA viruses (two phyla and over 70 classes) to those previously recognized by the ICTV (Neri et al. 2022).

Subsequently, a global sequence-based phylogenetic analysis was conducted, incorporating newly identified putative RdRP sequences from the Tara Oceans expedition (Zayed et al. 2022). In this analysis, consensus sequences were generated for each megataxon. If a consensus sequence contained more than 20% of ambiguous sites, the original sequence was used instead. The final phylogenetic tree was inferred using IQ-TREE with bootstrap support values (Zayed et al. 2022). This study reaffirmed the five branches (corresponding to the phyla assigned by the ICTV), with the exception of *Duplornaviricota*, which was split into three branches corresponding to the classes *Chrymotiviricetes*, *Resentoviricetes*, and *Vidavervicetes*, and interpreted as separate phyla (Zayed et al. 2022).

In addition to profile-based approaches based on aligned sequences, structure-based phylogenies have been developed for comparing distantly related proteins. It is well established that structural similarities between proteins are often more apparent than identifiable homologies within their underlying amino acid sequences (Chothia and Lesk 1986). This pattern is also evident among RdRPs, where detectably homologous sequence motifs tend to be shorter than their corresponding structural homomorphs (Lang et al. 2013). Consequently, structural conservation can provide insights into much deeper evolutionary history than what can be reliably resolved using sequence information.

The first structure-based phylogeny of viral RdRPs was inferred by Černý et al. (2015) using a manually created structural character matrix and sequence alignments. Subsequently, the structural relationships of viral RdRPs and related proteins have been revealed by comparing twenty physical and chemical properties using the Homologous Structure Finder (HSF) (Mönttinen et al. 2014, 2016, and 2021), by constructing hierarchical Cα atom superpositions using Theseus maximum-likelihood alignment (Peersen 2019), or by using structural alignment scores calculated from a pairwise comparison of root-mean-square deviation (RMSD) values and the number of superimposed residues (Jácome et al. 2015, 2022). In addition, Foldseek scores based on the 3D and amino acid substitution matrices (FoldTree software; Moi et al. 2025) have been recently applied to compare RdRPs of flavi- and flavi-like viruses within the order *Amarillovirales* as part of a major reclassification of the family *Flaviviridae* (Simmonds et al. 2025). FoldMason is the latest development in large-scale multiple structure alignment that can support phylogenetic analysis of conserved protein structures (Gilchrist et al. 2026). However, it has not been implemented for RdRPs yet. Recent studies have demonstrated the advantages of structure-based methods over traditional sequence-based phylogenetic approaches. For example, structure-based neighbor-joining trees have shown superior taxonomic congruence and ultrametricity compared to those based on amino acid sequences alone (Mifsud et al. 2025; Moi et al. 2025). Additionally, structure-guided alignments have proven more effective at detecting distant sequence patterns than conventional alignment methods (Mifsud et al. 2025). These findings highlight the potential of structural information in resolving deep evolutionary relationships.

The major limitation for broader structure-based phylogenetic analyses of orthornaviruses has been the small number of experimentally determined protein structures of viral RdRPs; these are typically only available for clinically important RNA virus pathogens, such as polio- and flaviviruses, or some well-established model systems (e.g. cysto- and fiersviruses). Thus, there is a glaring lack of comparative structural information for the much wider range of documented RNA viruses infecting arthropod, plant, fungal and bacterial hosts. However, this issue can now be alleviated through improved protein structure prediction methods.

In addition to scarcity of structural information, the interpretation of protein phylogenies inferred directly from structural similarities has been limited due to the lack of widely accessible methods for assessing the branch support for the structure-based phylogenetic trees. To our knowledge, none of the current structure comparison tools offer a built-in option for calculating branch support values from the structure-based distance matrices (Ravantti et al. 2013; Moi et al. 2025; Gilchrist et al. 2026). However, this was addressed recently by using a hybrid approach where structural information was used to strengthen the bootstrap value of the sequence-based phylogenetic tree (Baltzis et al. 2025). Namely, multiple sequence alignment was applied to anchor the positional homology, and intramolecular Cα–Cα distance matrices were computed per replicate which were then combined with sequence-based trees to yield a composite bootstrap (“multistrap”). While this approach strengthens the interpretation of structure-based phylogenetic trees, it cannot be directly applied to methods that are solely based on structural information. Nevertheless, this development underlines the importance of integrating structural signal to phylogenetic analyses of proteins.

In this study, we used AlphaFold software (Jumper et al. 2021; Guo et al. 2022; Abramson et al. 2024) to predict RdRP structure models for viruses lacking an experimentally determined structure and inferred a structure-based phylogeny for a set of predicted RdRP structures representing 96 RNA virus genera using HSF (Ravantti et al. 2013). HSF enables automated structure comparison of diversity of proteins. It progressively merges the most similar structure of pairs and identifies their common structural core replacing the pair in subsequent steps. This process continues until all structures are merged into a single common core. This core defines the set of structurally equivalent residues that are used in the next step to calculate a similarity distance matrix which is converted into a structure-based phylogenetic tree (Ravantti et al. 2013). HSF enables thorough comparisons of proteins within a protein family, like RdRPs (Mönttinen et al. 2014, 2021), but also between protein families and even superfamilies (Mönttinen et al. 2016). This significantly increases the depth of protein phylogenies. An additional key advantage of this method is its ability to accommodate RdRPs with swapped palm domain sequences, such as those in the members of *Birnaviridae* and *Permutotetraviridae* (Mönttinen et al. 2014; 2021) that defeat conventional sequence-based alignment approaches unless the sequences are manually re-ordered. In the current study, we also introduce a jackknife-based support metric to quantify the robustness of the HSF-derived structure-based phylogenies after removing subsets of structurally equivalent residues. Moreover, we made comparable analyses for the same dataset using FoldTree and FoldMason alignments. For the latter, we applied maximum likelihood tree that was based on the sequence information of the aligned structural residues and calculated bootstrap values to support phylogenetic interpretations.

Our findings demonstrate that AlphaFold-predicted structural models can be used reliably for structure-based phylogeny inference, provided they undergo a thorough quality validation. The common structural core, identified using HSF for 96 predicted RdRP structures representing members of 89 RNA virus families and seven floating genera, comprises 211 structurally equivalent residues, comparable to the core previously obtained for a smaller set of experimentally solved structures. This core covers ∼52% of the residues of the smallest RdRP structures in our dataset. Across three methods (HSF, FoldTree, and FoldMason), several well-established higher-level relationships were consistently recovered, including the strong monophyly of *Negarnaviricota* and most recognized class-level groups. At the same time, specific lineages such as classes *Duplopiviricetes*, *Pisoniviricetes*, *Magsaviricetes*, and the phylum *Pisuviricota* assemblage exhibited unstable or non-monophyletic placements, and *Flaviviridae* associated with *Kitrinoviricota* only under some inference settings. Thus, our results deviate from the previous sequence-based analysis and highlight the need of integrating the structural and sequence information to resolve deep phylogenetic relationships between RdRPs and to use that information to refine their taxonomic assignment.

## Results

### Prediction, selection and validation of RdRP structure

We selected 989 RdRP sequences representing diverse genera across 111 RNA virus families, along nine floating genera either assigned to the phylum *Lenarviricota* or unassigned to any phylum within the kingdom *Orthornavirae*, based on the classification recognized by the International Committee on Taxonomy of Viruses (ICTV Master Species List, release 39; https://ictv.global/msl) (see Table S1). We used AlphaFold2 to predict the corresponding protein structures (Supplementary Data). Following the quality control step, 814 high-confidence RdRP structures were retained (Table S2) representing 89 virus families, and seven virus genera not assigned to any family. Some of the included viral families are not fully classified at higher taxonomic ranks. One is a floating family assigned to the realm *Riboviria* (family *Polymycoviridae*), two to the kingdom *Orthornavirae* (families *Birnaviridae* and *Permutotetraviridae*), one to the phylum *Pisuviricota* (family *Hadakaviridae*), and one to the order *Yadokarivirales* (family *Yadokariviridae*), which is a floating taxon under the phylum *Pisuviricota*. Six of the floating genera belong to *Leviviricetes* class, and one is assigned to the kingdom *Orthornavirae* (*Botybirnavirus*; recently reclassified under the order *Chrymotiviricetes*).

To evaluate how accurately AlphaFold2 reproduces RdRP structures, we first compared a set of predicted structures with available experimental RdRP structures. We selected from the Protein Data Bank (PDB) experimental structures of 39 RdRPs representing 21 RNA virus families and 39 genera (Table S3). For each of these structures, we identified from our dataset a corresponding high-quality predicted RdRP structure from the same genus (Table S2 and Supplementary Data). If no such match was available within a genus, we selected a representative from a different genus within the same family, to maximize family-level coverage. Pairwise comparisons between the predicted and experimental structures were performed using sequence alignment, and the structures were then superimposed based on this information. The measured average RMSDs between pruned residues were 0.87 ± 0.25 Å (for all residues 3.75 ± 4.59 Å) indicating that the folds of the predicted structures closely follow the folds of the closely related experimentally solved RdRP structures justifying the use of predicted structures for the following structure-based phylogenetic analyses of RdRPs.

To evaluate the sensitivity of our RdRP predictions to the reliance of AlphaFold2 on the evolutionary information from multiple sequence alignments or structural templates, we predicted structural models for the set of 39 RdRP sequences also with the AlphaFold3 (Abramson et al. 2024) using the “no template’ option and compared the AlphaFold2 and template-free AlphaFold3 predictions. The average RMSD between AlphaFold2 and AlphaFold3 models was 2.09 ± 3.04 Å indicating that both methods recover the same overall RdRP fold.

To further assess the suitability of AlphaFold2-predicted structures for comparative and phylogenetic analysis of RdRPs, we evaluated how structural distances correlate with sequence identity. For this, we calculated pairwise RMSD values for the 39 AlphaFold2-predicted RdRP structures in our benchmark dataset and compared these with corresponding pairwise sequence identities. For sequence identities of 20% or greater, RMSD decreased approximately linearly with increasing sequence identity, and a fitted regression (*R*^2^ = 0.651) captured this trend (Fig. S1). This pattern is consistent with the well-known protein sequence–structure relationship in twilight zone (20% to 35% sequence identity), below which homology cannot be reliably inferred from sequence alone (e.g. Rost 1999). These results show that the AlphaFold2-predicted RdRP structures follow the expected relationship between sequence similarity and structural divergence, suggesting that the structural distances likely reflect the evolutionary relationships rather than prediction artifacts.

### Predicted RdRP structures exhibit a larger common structural core compared to experimentally solved structures

The 39 experimental RdRP structures and their corresponding 39 AlphaFold2- or AlphaFold3-predicted models were structurally aligned separately using HSF (Tables S3 and S4). A common structural core of 125 residues (average RMSD: 4.92 Å) was identified for the experimental structures, whereas the AlphaFold2- and AlphaFold3-predicted structures shared 249 (RMSD: 4.24 Å) and 171 (RMSD 5.09 Å) structurally equivalent residues, respectively. The smaller size of the common structural core identified for the experimental structures likely reflects unresolved regions and potential differences in structural conformation due to bound ligands.

#### Phylogenetic trees based on experimental and predicted structures share similar topologies

**Figure 1:**
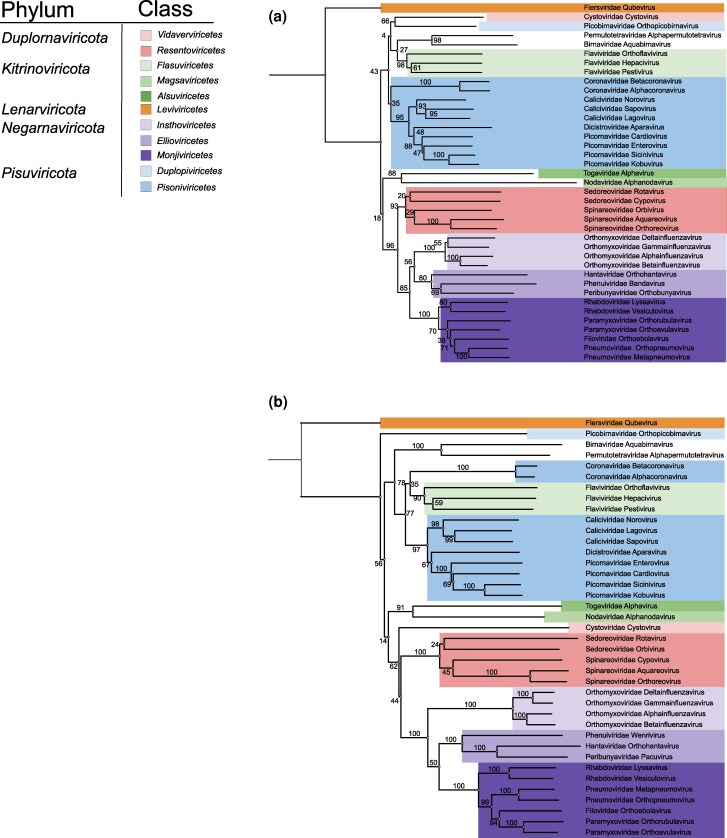
Structure-based phylogenetic trees produced using HSF for experimentally solved and AlphaFold2-predicted RdRP structures. (a**)** Neighbor-joining tree based on experimentally solved RdRP structures from 39 RNA viruses. (b**)** Corresponding trees based on AlphaFold2-predicted RdRP structures that have been matched taxonomically to the experimentally solved structures. The taxa are colored by taxonomic class, and the explanations for color coding are shown on top left. The jackknifing support values are shown for the branches.

Structure-based phylogenetic trees were deduced by automatic structural comparison of the equivalent residues of the identified common structural cores for the 39 experimental and predicted RdRP structures. The trees were rooted using the *Leviviricetes* (bacterial ssRNA viruses) as the outgroup. According to previous studies, RdRPs of levivirids have diverged early from the common ancestor of viral RdRPs (de Farias et al. 2017; Wolf et al. 2018). The resulting trees showed largely consistent topologies (Figs. 1 and S2). Comparison of the trees based on the predicted-structure with the tree derived from experimental structures yielded normalized Robinson–Foulds distances of 0.361 (AlphaFold2 vs. experimental) and 0.417 (AlphaFold3 vs. experimental). These moderate differences likely reflect the substantially smaller common structural core available among experimental structures, which provides a weaker phylogenetic signal relative to the larger common cores of the predicted models. The normalized Robinson–Foulds distance between the phylogenetic trees based on the AlphaFold2- and AlphaFold3-predicted structures was only 0.167 indicating that the use of template information in the structure prediction does not markedly influences the inferred relationships.

### Structure-based jackknifing method to measure tree robustness

To assess the sensitivity of the structure-based tree topology to perturbations in the common structural core identified by HSF, we developed a jackknifing method that randomly samples equivalent sites across studied structures. An appropriate sampling fraction was identified by randomly selecting the individual equivalent sites within the common structural core. Sampling fractions from 10% to 90% were evaluated, and for each level 100 jackknife replicates were generated. Robinson–Foulds values decreased steeply at low sampling and entered a region of diminishing change at ∼40% (Fig. S3). Based on this stabilization region, we selected 50% sampling for jackknife analysis. Because structural residues exhibit spatial correlation, we next assessed how random-site versus block-based sampling affect tree topology at the 50% level. Block-based removal systematically yielded higher normalized Robinson–Foulds distances than removal of random residues (0.39 ± 0.062 vs. 0.31 ± 0.064), indicating stronger perturbation of the structural signal. We therefore used contiguous block-based random sampling in the final jackknife procedure, selecting random blocks ranging from 10 residues up to the maximum allowable sampled block size. A total of 100 replicates were generated, and the resulting jackknife support values were mapped onto the tree based on the full core.

#### Phylogenetic trees based on predicted structures demonstrates strong jackknife support

The structure-based jackknife method was used to evaluate the stability of the trees produced from the 39 experimental and AlphaFold-predicted structures using HSF. Notably, the trees based on AlphaFold2- and AlphaFold3-predicted structures demonstrated stronger jackknife support than the tree based on experimental structures with mean (median) supports of 70.7 (80) for experimental structures, 81.9 (98.5) for AlphaFold2, and 76.7 (95.5) for AlphaFold3 structures (Figs. 1 and S2). This likely reflects the larger structural cores of the predicted structures, which makes them more resistant to noise compared to the trees produced using the smaller common structural core of the experimental structures. Thus, the additional regions in the common cores of the predicted structures likely reflect homologous, evolutionarily conserved features rather than random similarities. These findings support the use of high-quality predicted structures in large-scale phylogenetic analyses.

Overall, the three trees were largely congruent, and the groupings were broadly consistent with existing RdRP sequence-based phylogenies (Wolf et al. 2018). However, the placements of picobirnavirus (member of the phylum *Pisuviricota*), cystovirus (phylum *Duplornaviricota*), and members of the *Flaviviridae* (phylum *Kitrinoviricota*) showed inconsistencies among the trees and were not fully aligned with their current taxonomic classification (Figs. 1 and S2).

#### The structure-based phylogeny of viral RdRPs largely supports class-level classification of RNA viruses

**Figure 2:**
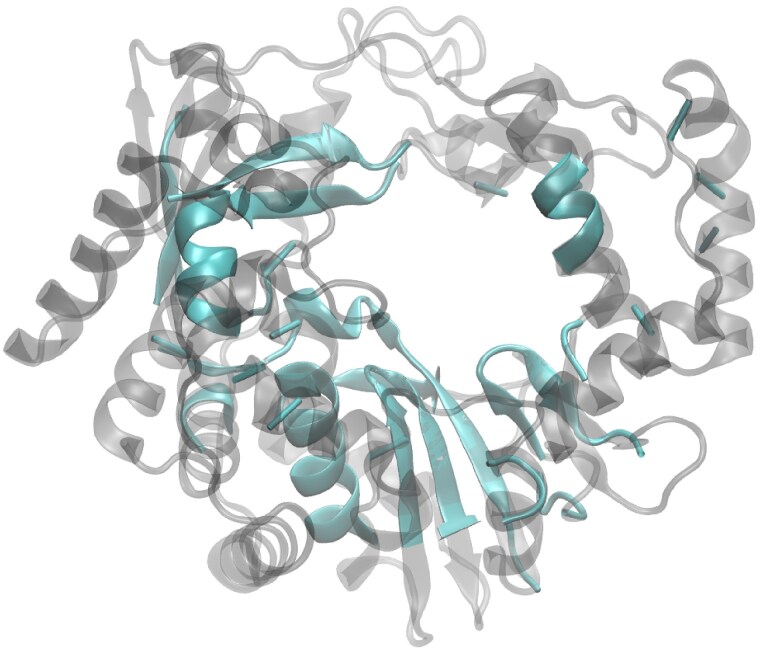
The common structural core depicted on the structural model of crohivirus RdRP. The 211 equivalent residues identified using HSF for the 96 predicted RdRP structures (green) depicted on the smallest RdRP in the data set (structural model of the crohivirus RdRP; light gray).

A structure-based comparison using HSF was next performed on 96 high-quality predicted RdRP structures, with each structure representing either one of the 89 recognized RNA virus families or one of the seven floating genera included on our dataset (Table S5 and Supplementary Data). Together, this dataset includes RdRPs from 13 bacterial, 18 fungal, 14 plant, 3 algae, 1 protist and 47 vertebrate/invertebrate viruses (Table S5). A conserved structural core comprising 211 residues was identified for this diverse set of viral RdRPs (average RMSD: 4.0 Å; Fig. 2), primarily encompassing the palm and parts of the fingers subdomain covering ∼52% of the residues of the crohivirus RdRP which represents the smallest RdRP structures used in the analysis.

**Figure 3:**
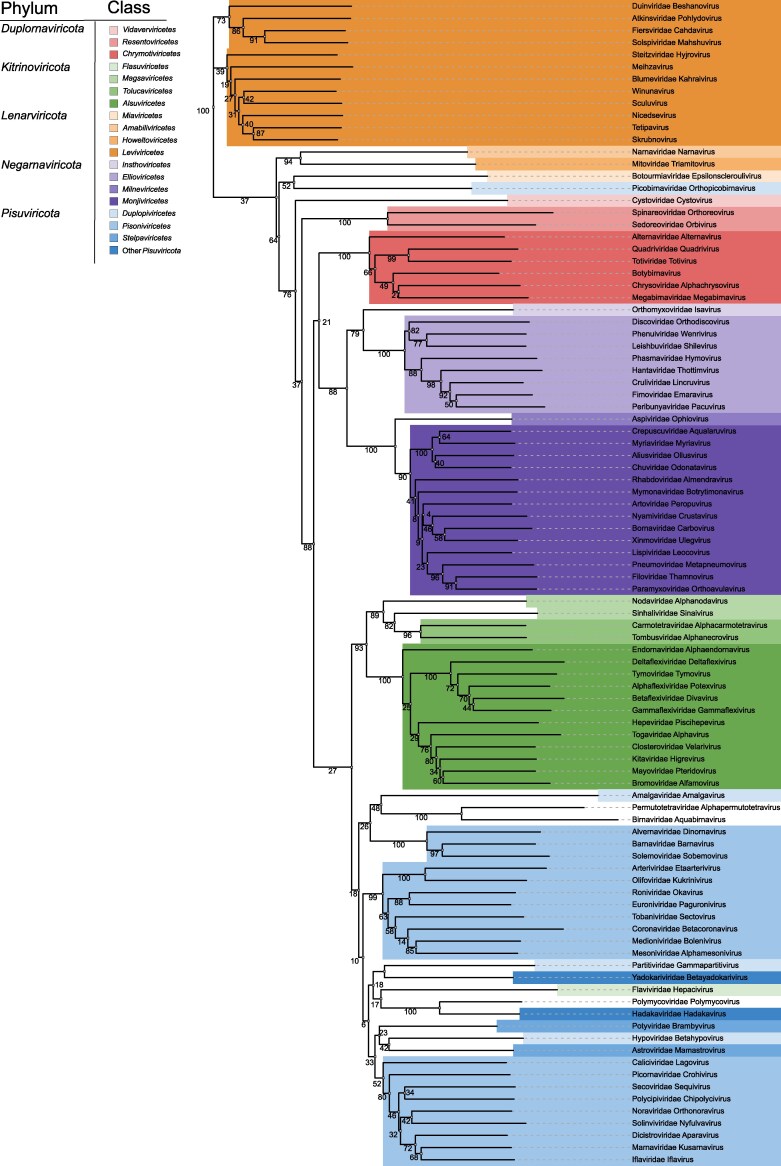
Structure-based phylogenetic tree for viral RdRPs produced using HSF. A full-scale neighbor-joining tree was inferred from 96 AlphaFold2-predicted RdRP structures, representing 89 virus families and seven floating genera. Taxa are color-coded by phylum and class, as indicated in the reference table in the top left corner. Jackknifing support values are shown at each node to indicate the reliability of each branch.

The comparison of the identified 211 equivalent residues between the 96 predicted structures (Fig. 2) resulted in a phylogenetic tree (Fig. 3) which robustly supports existing taxonomic assignments at the class rank. Strong (≥90%) jackknife support was obtained for the monophyly of seven classes (*Leviviricetes*, *Resentoviricetes*, *Chrymotiviricetes*, *Ellioviricetes*, *Monjiviricetes*, *Tolucaviricetes*, and *Alsuviricetes*), which were also well separated from the additional seven classes that in our dataset contained only one representative RdRP. However, the grouping of the viruses under the phylum *Pisuviricota* largely violated the currently assigned classes *Duplopiviricetes*, *Pisoniviricetes*, and *Stelpaviricetes*. Notably, the four families of the class *Duplopiviricetes* (*Partitiviridae*, *Amalgaviridae*, *Hypoviridae*, and *Picorbirnaviridae*) were highly scattered in our structure-based phylogenetic tree inferred using HSF. Moreover, the two orders of the class *Pisoniviricetes* (*Nidovirales and Sobelivirales*) made strongly supported monophyletic clades (branch support values 100 and 99, respectively), but these did not form a monophyletic group together at the class level. *Picornavirales*, the third order of *Pisoniviricetes*, also formed a monophyletic group, which is relatively strongly supported if *Caliciviridae* is excluded (branch support 80 without calicivirus; 52 including calicivirus). Furthermore, the class *Magsaviricetes* appears paraphyletic with respect to the monophyletic class *Tolucaviricates* within the phylum *Kitrinoviricota*.

### Support and violations of the phylum level classification

RdRPs from negative-strand (−)RNA viruses (phylum *Negarnaviricota*) formed a well-supported monophyletic clade in the structure-based phylogenetic tree based on AlphaFold2-predicted structures (Fig. 3). In contrast, members of the *Duplornaviricota* phylum formed three distinct and strongly supported clades corresponding to the classes *Vidavervicetes*, *Resentoviricetes*, and *Chrymotiviricetes*.

The members of the phylum *Kitrinoviricota* grouped strongly together (93% jackknife support), with the exception of family *Flaviviridae* which repeatedly clustered with the members of the *Pisuviricota* phylum. Majority of the members of the phylum *Pisuviricota* clustered together, although with low support. Instead of grouping with *Pisuviricota*, orthopicobirnavirus (family *Picobirnaviridae*, speculated to infect a bacterial host; Hutton et al. 2025) was placed among *Lenarviricota* members, which include bacterial (*Leviviricetes*) and mitochondrial (*Howeltoviricetes*) RNA viruses. However, its precise position as a sister group to botourmiaviruses (class *Miaviricetes*; including viruses infecting fungi, oomycetes and plants) received only weak support (52% jackknife), and therefore this placement should be interpreted with caution.

#### Associations of floating taxa with the existing taxonomy

The cluster comprising members of the *Pisuviricota* phyla (except orthopicobirnavirus), also contained the two floating families of the kingdom *Orthornavirae,* the *Birnaviridae* and *Permutotetraviridae,* which together formed a strongly supported clade. Furthermore, the floating family of the realm *Riboviria* (*Polymycoviridae*) was strongly associated with *Hadakaviridae* (a floating family of *Pisuviricota*), suggesting a shared ancestry. Moreover, the floating genera of the *Leviviricetes* class included in our dataset all clustered in a well-supported branch comprising currently recognized members of the order *Timlovirales*, and botybirnavirus RdRP associated strongly (100% jackknife support) with members of the class *Chrymotiviricetes*, supporting the current classification of family *Botybirnaviridae*. Yadokarivirus RdRP (family *Yadokariviridae*, floating class *Yadokarivirales*) associated with *Partitiviridae*.

#### Comparison to other cluster-based methods

To further validate our results, we applied FoldTree (Moi et al. 2025) and FoldMason (Gilchrist et al. 2026) software for the same set of 96 predicted RdRP structures used in the full-scale HSF-based RdRP comparison. FoldTree does not provide statistical support values for tree branching patterns, limiting the assessment of the robustness of individual nodes. However, it is useful for evaluating whether similar topological features emerge across different structure-based methods. For FoldMason, the sequences of structurally aligned residues were used to deduce a maximum likelihood tree and to calculate bootstrap values (see Methods).

**Figure 4:**
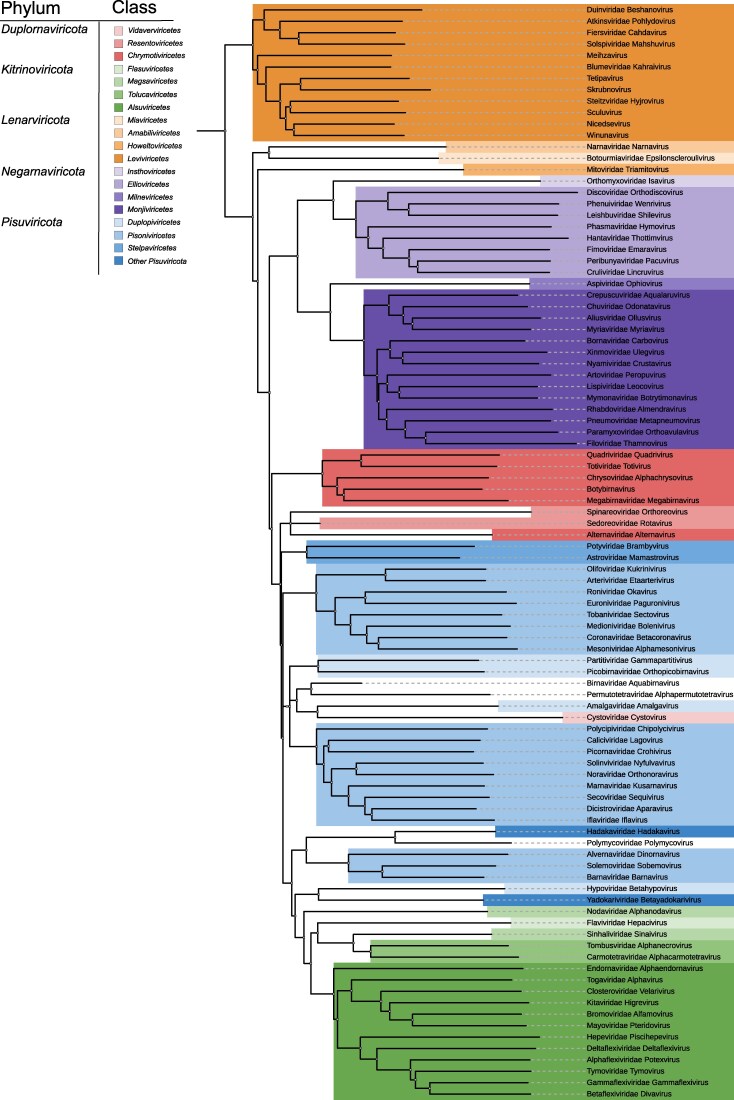
Structure-based phylogenetic tree for viral RdRPs inferred using FoldTree. The same set of 96 AlphaFold2-predicted RdRP structures were used as for the full-scale analysis with HSF (see Fig. 3). Taxa are color-coded by phylum and class, as indicated in the reference table in the top-left corner.

**Figure 5:**
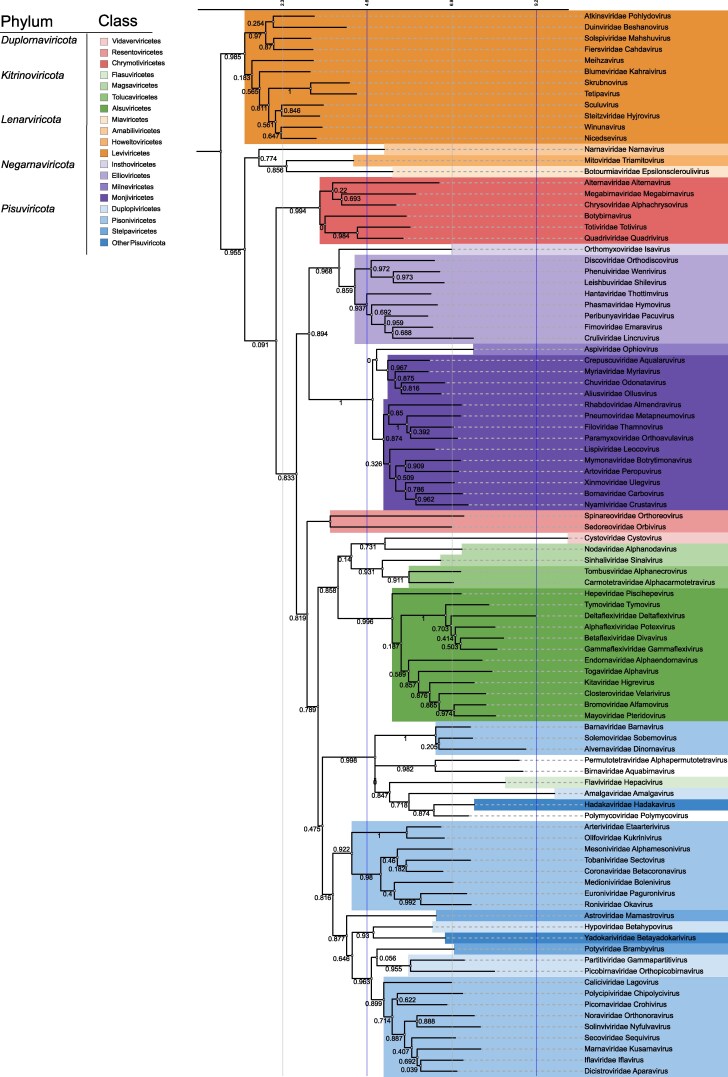
Structure-based phylogenetic tree based on FoldMason alignment. Branches are labeled with viral family and genus names and are colored according to the taxonomic class. The color legend is shown in the top-left corner. Bootstrap support values are indicated for the branches. The tree scale is shown above the tree.

The overall topologies of the structure-based phylogenetic trees inferred using FoldTree (Fig. 4), FoldMason (Fig. 5), and HSF (Fig. 3) were moderately similar in the tree topology (a normalized Robinson–Foulds distance for HSF and FoldTree was 0.486 and HSF and FoldMason 0.54). RdRPs of all (−)RNA viruses formed a single cluster in all three trees, and the branching of classes within *Negarnaviricota* was identical across method (Figs. 3–5). Members of *Duplornaviricota* formed three distinct clades in the FoldMason and HSF trees (Figs. 3 and 5). However, topological differences were observed for these dsRNA viruses: in the FoldTree-based analysis, *Vidaverviricetes* (family *Cystoviridae*) grouped apart from the other *Duplornaviricota* clustering together with dsRNA viruses of the *Pisuviricota* (i.e. with the class *Duplopiviricetes*) and in the FoldMason tree cystoviruses, representing bacterial dsRNA viruses, grouped with members of the phylum *Kitrinoviricota* comprising eukaryotic ssRNA viruses. Furthermore, FoldTree analysis suggested *Chrymotiviricetes* class to be polyphyletic (Fig. 4), while it forms a strongly supported monophyletic clade in both the HSF-based tree (100% jackknife support; Fig. 3) and the FoldMason tree (0.994 bootstrap value; Fig. 5).

All three software produced trees with three distinct branches comprising the RdRPs of *Lenarviricota* phylum. However, the branching patterns among narnaviruses, botourmiaviruses, and mitoviruses differed between the methods: the strongly supported narnavirus–mitovirus cluster seen in the HSF tree (Fig. 3) was not reproduced by FoldTree, although in the FoldMason tree all three lineages (narna-, botourmia-, and mitoviruses) grouped together with strong support (Fig. 5).

In both FoldTree and FoldMason trees, RdRPs from the members of *Pisuviricota* (including orthopicobirnavirus), *Kitrinoviricota,* as well as birna-, permutotetra-, and polymycoviruses (representing floating families of realm *Riboviria* and kingdom *Orthornavirae*) and cystoviruses (phylum *Duplornaviricota*) formed a single mixed cluster. As in the HSF analysis, the orders of the *Pisoniviricetes* class (i.e. *Nidovirales*, *Sobelivirales*, and *Picornavirales*) did not cluster together, indicating paraphyly of *Pisoniviricetes* across all methods. The class *Magsaviricetes* of the phylum *Kitrinoviricota* is also paraphyletic based on the structure-based phylogenetic trees (Figs. 3 and 4 and 5). Flaviviruses clustered with members of the *Pisuviricota* phylum in both the HSF and FoldMason trees (Figs. 3 and 5), where only the FoldMason tree provided high support. In contrast, the FoldTree grouped flaviviruses with the members of the phylum *Kitrinoviricota* (Fig. 4).

All three approaches supported a shared origin of polymycoviruses and hadakaviruses (strongly supported by both HSF and FoldMason trees). Likewise, RdRPs of birna- and permutotetraviruses—representing viruses with a permuted palm domain—clustered together across all trees. Their closest relatives in the structure-based trees appeared to be *Pisuviricota*-like viruses, although this association received strong support only in the FoldMason tree.

## Discussion

In this study, we evaluated the suitability of AlphaFold-predicted protein structures for structure-based phylogenetic analyses of viral RdRPs. Structure-based phylogenies offer a valuable complement to sequence-based methods, particularly when sequences are divergent and problematic to align, as in the case of RdRP sequences of RNA viruses in the kingdom *Orthornavirae*. Previously, the application of structure-based phylogenies has been limited by the bias of experimentally solved RdRP structures. As a result, RdRPs from viruses infecting plants, fungi, insects, and bacteria have been underrepresented or entirely omitted.

The advent of AlphaFold offers a promising solution to this limitation by enabling high-confidence structural predictions across a broader taxonomic range. To assess the impact of predicted structure quality on phylogenetic inference, we conducted two analyses: (i) A comparative structure-based phylogeny using corresponding sets of both experimental and predicted structures (Figs. 1 and S2), and (ii) a comprehensive structure-based phylogeny including 96 high-quality predicted structures representing members of 89 RNA virus families and seven floating genera (Fig. 3). Furthermore, we compared RdRP structures predicted with two different methods (AlphaFold2 and AlphaFold3; Figs. 1b and S2) as well as three different structure comparison approaches based on HSF, FoldTree, and FoldMason (Figs. 3–5). Notably, we introduced jackknifing support values for branches of structure-based phylogenetic trees that do not require converging structural alignment into a sequence alignment.

The topologies of phylogenetic trees derived from experimental and predicted structures were generally consistent at higher taxonomic levels. However, the trees based on AlphaFold-predicted structures had stronger jackknifing support than the tree based on experimental structures, suggesting that unresolved regions, represented as gaps in experimental solved structures, may compromise the robustness of the tree. Because HSF aligns structures progressively (Ravantti et al. 2013), missing regions in initial alignments remain through subsequent rounds, reducing the size of the common structural core. This effect is amplified in large trees, where the cumulative gaps can significantly impact the phylogenetic tree resolution. However, it should be noticed that AlphaFold-predicted structures can include low-confidence regions (i.e. predicted local difference distance test [pLDDT] < 50), which may be structurally unreliable. Therefore, we thoroughly pre-processed the predicted structures to identify and exclude such regions (or poor-quality models). This is essential to ensure the accuracy and robustness of structure-based phylogenies.

Our full-scale phylogenetic tree based on 96 predicted RdRP structures and deduced using HSF, FoldMason or FoldTree (Figs. 3–5) broadly supports the current RNA virus taxonomy which is based on comparison of RdRP sequences (Wolf et al. 2018). However, discrepancies were observed both at the class and phylum levels. In particular, our findings do not support the monophyly of *Duplopiviricetes*, a finding consistent with previous reports (Neri et al. 2022) that identified orthopicobirnavirus as a frequent violator of established phylum-level classifications.

Our results also suggest that the *Duplornaviricota* phylum is not monophyletic but rather is separated to three distinct clusters that correspond to the *Vidaverviricetes*, *Resentoviricetes*, and *Chrymotiviricetes* classes. This pattern mirrors the findings of Zayed et al. (2022) and further supports the need for a taxonomic revision. Similarly, *Cystoviridae*, a member of *Duplornaviricota* phylum, was identified as a frequent violator of phylum-level classification in the phylogenetic analysis of Neri et al. (2022).

Our results supported the monophyly collectively of the *Kitrinoviricota* and *Picornaviricota* phyla, a grouping that includes *Birnaviridae*, *Permutotetraviridae* and *Polymycoviridae* (Figs. 3–5). However, despite of consistent grouping, it received high support only in FoldMason-based tree. The exception is the placement of *Cystoviridae* (phylum *Duplornaviricota*) within this group by FoldTree and FoldMason (Figs. 4 and 5). The monophyly of the *Pisuviricota* is poorly supported—contradicting earlier studies that supported its monophyly (Wolf et al. 2018). In the HSF and FoldMason analyses, *Kitrinoviricota* was strongly monophyletic (support values 93 and 0.858, respectively; Figs. 3 and 5) with the exception of the *Flaviviridae* that repeatedly violates the established classification regardless of the origin of the structures (experimental as well as AlphaFold2- and AlphaFold3-predicted structures; Figs. 1 and S2) or the structural comparison software (HSF and FoldMason). Nevertheless, this violation was not observed in the FoldTree-based tree, suggesting some method-dependent variation in the placement of *Flaviviridae*. Notably, Neri et al. (2022) also identified *Flaviviridae* as one of the most frequent violators of established taxonomy, potentially reflecting its divergent or complex evolutionary history.

The class *Pisoniviricetes* appeared polyphyletic in all our analyses—including HSF, FoldMason and FoldTree trees based on AlphaFold2-predicted structures (Fig. 1b, 3–5), as well as the HSF trees based on experimental and AlphaFold3-predicted structures (Figs. 1a and S2). Nevertheless, the three orders of *Pisoniviricetes* class (*Nidovirales*, *Sobelivirales*, and *Picornavirales*) formed monophyletic groups. Furthermore, the monophyly of *Pisuviricota* phylum was not supported, and flaviviruses from *Kitrinoviricota* phylum were frequently placed within the cluster of *Pisuviricota* members (Figs. 3 and 5), while picobirnaviruses were placed separately from the other *Pisuviricota* members in HSF-based trees (Figs. 1, 3, and S2).

Structure-based phylogenetic methods have been criticized for their non-additive nature, which may limit their effectiveness in identifying deep evolutionary relationships (Edgar 2023). However, we would argue that these methods complement sequence-based approaches, which are affected by recombination, inversions, rate heterogeneity, and, for some viruses in the current dataset, domain swaps that preclude the use of unsupervised alignment methods. Furthermore, based on our analysis, there is a linear relationship between structural similarity (RMSD) and sequence identity above the twilight zone, indicating correlation between the evolutionary signals in 3D structures and sequences (Fig. S1). Although the potential convergent evolution of folds and the absence of a unified phylogenetic model remain challenges, the HSF software used in this study incorporates amino acid properties and secondary structure features (Ravantti et al. 2013), thereby reducing the risk of interpreting convergent folds as homologous. Moreover, the three different structure-based methods used in this study provided roughly similar results and support for the robust parts of the tree, suggesting that structure-based software can capture evolutionary signals.

Importantly, our structure-based phylogenies show broad agreement with the profile-based sequence similarity trees (Wolf et al. 2018; Neri et al. 2022). The problematic taxa identified in our analysis overlap with those reported by Neri et al. (2022) and Zayed et al. (2022), reinforcing the validity of our findings. Jackknifing support values further emphasize the need for caution when interpreting deep phylogenies of RdRPs. Profile-based sequence similarity trees are also subject to limitations including highly variable sites, short alignments, and the variation in amino acid frequency across the dataset, which complicates the application of substitution models and the interpretation of deep phylogenies applied (Holmes and Duchêne 2019).

In summary, our findings demonstrate that AlphaFold-predicted structures can effectively be used to infer phylogenetic relationships. This serves as a valuable complement to, and sequence-alignment-free alternative for, sequence-based analyses. While model quality remains critical—since even a single low-confidence region can distort tree topology—predicted structures offer the advantages of being gap-free and widely available across diverse taxa. Current structure-based methods rely on distance metrics, which are susceptible to long-branch attraction (Felsenstein 1981). Developing probabilistic models similar to those used in sequence-based phylogenetics could allow maximum likelihood methods to be applied, thereby improving the placement of distantly related RdRPs.

The challenge of inferring deep evolutionary relationships for rapidly evolving RNA viruses requires a combination of different methodologies to identify common features in the phylogenies. The accumulating data on structure- and sequence-based phylogenetic analyses should direct the revision of the current RNA virus classification to better reflect the likely evolutionary relationships.

## Materials and methods

### Selection of representative viral RdRP genes

Exemplar viruses of all RNA virus genera, recognized by ICTV (before April 2023), were collected from the Virus Metadata Resource spreadsheet (ICTV classification table; see Table S1). RefSeq nucleotide identifiers were subsequently collected for each selected virus, or GenBank identifiers if RefSeq identifiers were not available. Using the Entrez Python package (https://biopython.org/docs/1.75/api/Bio.Entrez.html) (Cock et al. 2009), the corresponding NCBI nucleotide entry files were downloaded, and their “product” or “matpeptide” fields were screened using a set of established RdRP names (e.g. replicase, RdRP, L protein, nsp12, 3D, NIb, VP1, PB1, NS5). If an RdRP name was found in the product or “matpeptide” field, the amino acid sequence was parsed directly from the nucleotide entry file or downloaded through the Entrez interface, respectively. If the sequence was >3,000 amino acids, the sequence was screened for motifs A–C, ±1,000 amino acids were added around the identified region, and the sequence was trimmed accordingly.

If no RdRP name was found from these fields, all NCBI protein entries for each annotated open reading frame (ORF) of the virus were downloaded from Entrez, and the Region_Name field of these entries was searched for the RdRP names. If such a name was found, the note fields were searched for A, B, C, D, E, and F motifs (Bruenn 2003). The smallest and largest motif indexes were extracted with additional amino acids at both ends to cover the full RdRP estimated based on Bruenn (2003). If there were no annotations for the motifs, the ORFs were manually searched with BLASTp (Priyam et al. 2019) against the NCBI's RefSeq database, and the smallest and largest indexes for RdRP motif prediction with extra amino acids estimated based on Bruenn (2003) at both ends were extracted. No ORFs were annotated for two of the selected example viruses (Entrez identifiers: GBBW01007738.1 and GECV01031551). These sequences were analyzed using BlastX (Priyam et al. 2019) to identify a putative RdRP region, which was then cleaved from the sequence and translated using the ExPASy tool (https://web.expasy.org/translate/; Gasteiger 2003).

In the final step, one representative sequence per genus was chosen. Uncut sequences without unknown amino acids were preferred. If such sequences were not available, a sequence was trimmed so that the unknown amino acids, which in all cases were located at the termini of the sequence, were removed. In total, 23 sequences were affected (Table S6).

The experimentally solved RdRP structures were collected from the PDB (on the 2025 February 28) using a combination of query words. When selecting the experimental structures, we ensured that each one had an equivalent from the same virus genus among the high-quality predicted structures (Table S3).

### Structure prediction, quality control and trimming of the predicted RdRP structures

The RdRPs were predicted using AlphaFold v.2.3.1 (Guo et al. 2022) with the databases updated on the 2023 August 1. The AlphaFold was used through the Singularity interface and each structure prediction was given 1 GPU, 8 CPU, and 90G of memory. For comparison, RdRPs were predicted also with Alphafold v.3.0.1 (Abramson et al. 2024) in the Google DeepMind web user interface (www.alphafoldserver.com). All settings were set to default with the exception of “templates,” which was disabled.

The pLDDT (Mariani et al. 2013; Guo et al. 2022) scores of each AphaFold-predicted RdRP structure were screened as part of the quality control process. At the N- and C-termini, residues were screened with a 15-amino acid window, and the spot where all residues within the window received a pLDDT score above 70 was identified. The residues before this spot at the N-terminus were deleted, as were the residues after the identified spot at the C-terminus.

The percentage of residues receiving pLDDT scores below 50 and below 70 in the remaining structure was then calculated. Structures were not selected for further analysis if the percentage of residues with pLDDT scores below 50 was higher than 5%, or if the percentage of residues with pLDDT scores below 70 was higher than 10% (Tables S1 and S2).

One structure from each virus family was selected for further analysis. Structures with the highest average pLDDT scores were preferred. Before inferring the structure-based phylogeny, residues with a pLDDT score of less than 50 were removed from the selected structures (Table S5). Furthermore, the largest structures were trimmed prior to the structure-based phylogeny analysis. To define the relevant regions, the indexes for the RdRP domains were identified again from the NCBI protein entries using a set of keywords (e.g. *RNA-dependent*, *RdRp*, *RNA-directed*), and 200 amino acids were added to both ends. If a structure exceeded this range, the regions outside the adjusted indexes were trimmed accordingly. If there was no such annotation in the NCBI protein entry, the structure was not trimmed.

#### RMSD distances

The RMSD distances between experimental and AlphaFold-predicted structures were calculated with Matchmaker from Chimerax (Pettersen et al. 2021) with parameters: Chain pairing bb, Alignment algorithm: Needleman-Wunsch, Similarity matrix: BLOSUM-62, SS fraction: 0.3, Gap open (HH/SS/other): 18/18/6, Gap extend: 1, SS matrix: 6, −9, −6; 6, −6; 4, and Iteration cutoff: 2. Conversely, RMSD distances for all-verses-all comparisons of AlphaFold2 and AlphaFold3 structures were calculated using Foldseek v.10.941cd33 (van Kempen et al. 2024), using default settings in easy-search mode.

#### Structure-based phylogeny

The RdRP structures were aligned and their equivalent residues identified using HSF (Ravantti et al. 2013). The parameters previously optimized for right-hand-shaped polymerases were applied in this study (Mönttinen et al. 2016; 2021). HSF employs progressive pairwise comparison of structures to identify equivalent residues. The common cores identified for pairs are subsequently aggregated into groups (subcores). These subcores are then merged into a final core comprising the structurally equivalent residues present in all the structures in the dataset. This approach culminates in the identification of equivalent residues for the entire dataset, as well as for various subsets (subcores) that exhibit elevated levels of similarity.

The structure-based distance tree was deduced based on the identified equivalent residues shared by all the structures in the dataset, that is, the common structural core. Pairwise scores were initially calculated using the core residues. In the absence of established methodologies for converting structural alignments into distances that reflect evolutionary trajectories, we have opted to employ the HSF approach (Ravantti et al. 2013) to convert the obtained scores into distances. The distance between two structures, A and B, is calculated using the pairwise score, S(A,B), as follows: D(A,B) = −(S(A,B) − min[S(A,A), S(B,B)]). The all-against-all distance matrix was converted to a tree using Quicktree (Howe et al. 2002).

A structure-based phylogeny was also inferred using FoldTree (Moi et al. 2025) with the default parameters. The FoldMason (Gilchrist et al. 2026) alignment was performed with default settings. The tree was inferred using the maximum-likelihood method in Fasttreev2.0.0 (Price et al. 2010) with 1,000 bootstrap iterations.

### Jackknifing for HSF-based trees

Jackknifing was performed for the HSF-based trees by sampling the equivalent residues of the common structural core 100 times. First, the individual residues were selected at random each time, and then in blocks. The length of these blocks varied randomly between ten and of the maximum number of residues sampled. For each replicate, the distances between structures were recalculated as described in the “Structure-based phylogeny’ section, and phylogenetic trees were inferred using QuickTree (Howe et al. 2002). The jackknifing support values for the original phylogenetic tree were mapped using IQ-TREEv2.1.4 (Minh et al. 2020) with the command “iqtree -sup reference_tree replicate_trees -pre prefix’.

To assess the sensitivity of the phylogenetic reconstruction to the amount of structural information used, a sweep test was performed in which 10%, 20%, 30%, 40%, 50%, 60%, 70%, 80%, and 90% of the residues from the common structural core were sampled. For each sampling level, 100 replicate datasets were generated, and phylogenetic trees were inferred as described in “Structure-based phylogeny’ section.

Each tree within a replicate set was compared with the reference tree using the Robinson–Foulds distance, providing a quantitative measure of topological deviation. To evaluate the structural disturbance introduced by sampling, the average Robinson–Foulds distance was calculated using Python EteToolkit v3.1.3 (Huerta-Cepas et al. 2016), and the relationship between sampling proportion and Robinson–Foulds distance was visualized in a summary plot.

### Tree comparisons

Trees were compared using Robinson–Foulds distance that was calculated using Python EteToolkit v3.1.3 (Huerta-Cepas et al. 2016).

### Visualization

All trees were visualized with iTol (Letunic and Bork 2024). The common structural core of RdRPs was visualized with Visual Molecular Dynamics v2.0.0a5 (Humphrey et al. 1996).

## Supplementary Material

msag088_Supplementary_Data

## Data Availability

The Python code for data processing and analyses is available at https://github.com/HeliMonttinen/RdRp_models. The data underlying this article is available at https://etsin.fairdata.fi service https://doi.org/10.23729/fd-d675f457-9961-32fe-b2d3-39ee2eae59a0. All other data are included in the manuscript and/or supporting information.
