# Supplementary material for: Revealing deep evolutionary relationships between RNA viruses using predicted structural models of viral RNA polymerases
Source: Mol Biol Evol. 2026 Apr 7;43(4):msag088. doi: 10.1093/molbev/msag088 (PMC13107431; doi:10.1093/molbev/msag088)
Supplement: msag088_Supplementary_Data [file msag088_supplementary_data.zip › Supplementary_Figures.pdf]

Supplementary Figure S1.

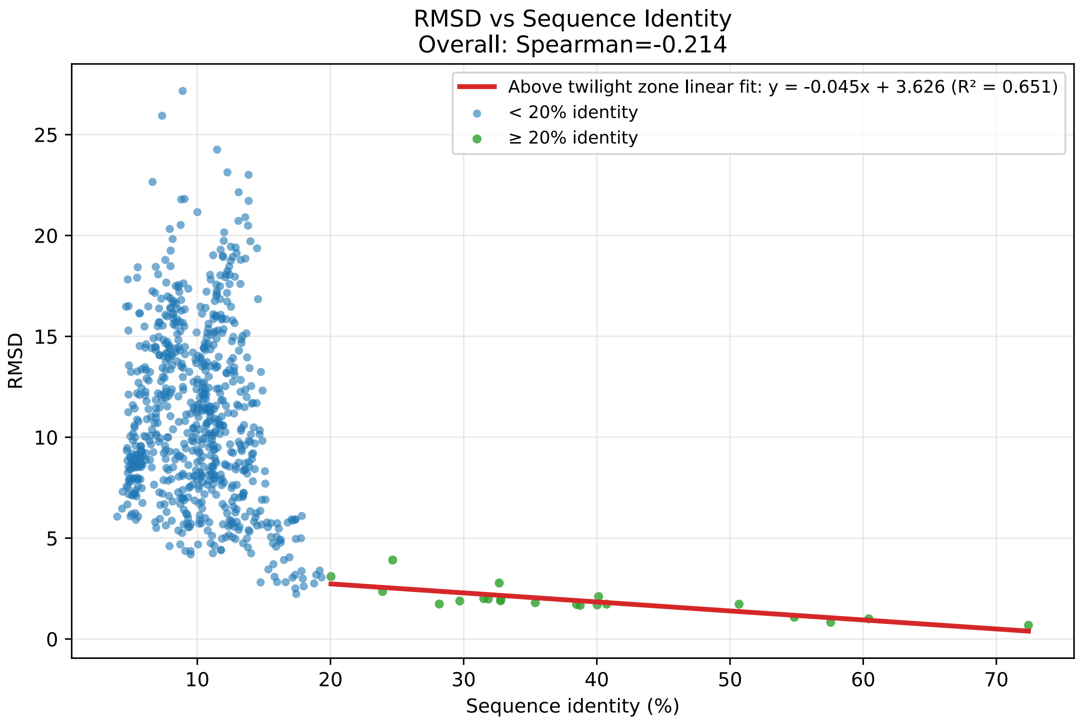

Supplementary Figure S1. Pairwise sequence identity versus structural distance. All AlphaFold2-predicted structures were compared pairwise, and RMSDs of the aligned structures were calculated together with their sequence identities. Each point represents the RMSD of a structure pair plotted against its sequence identity. A linear regression model was fitted for pairs with sequence identity above 20%. Spearman correlation coefficients were calculated for the full dataset.

Supplementary Figure S2

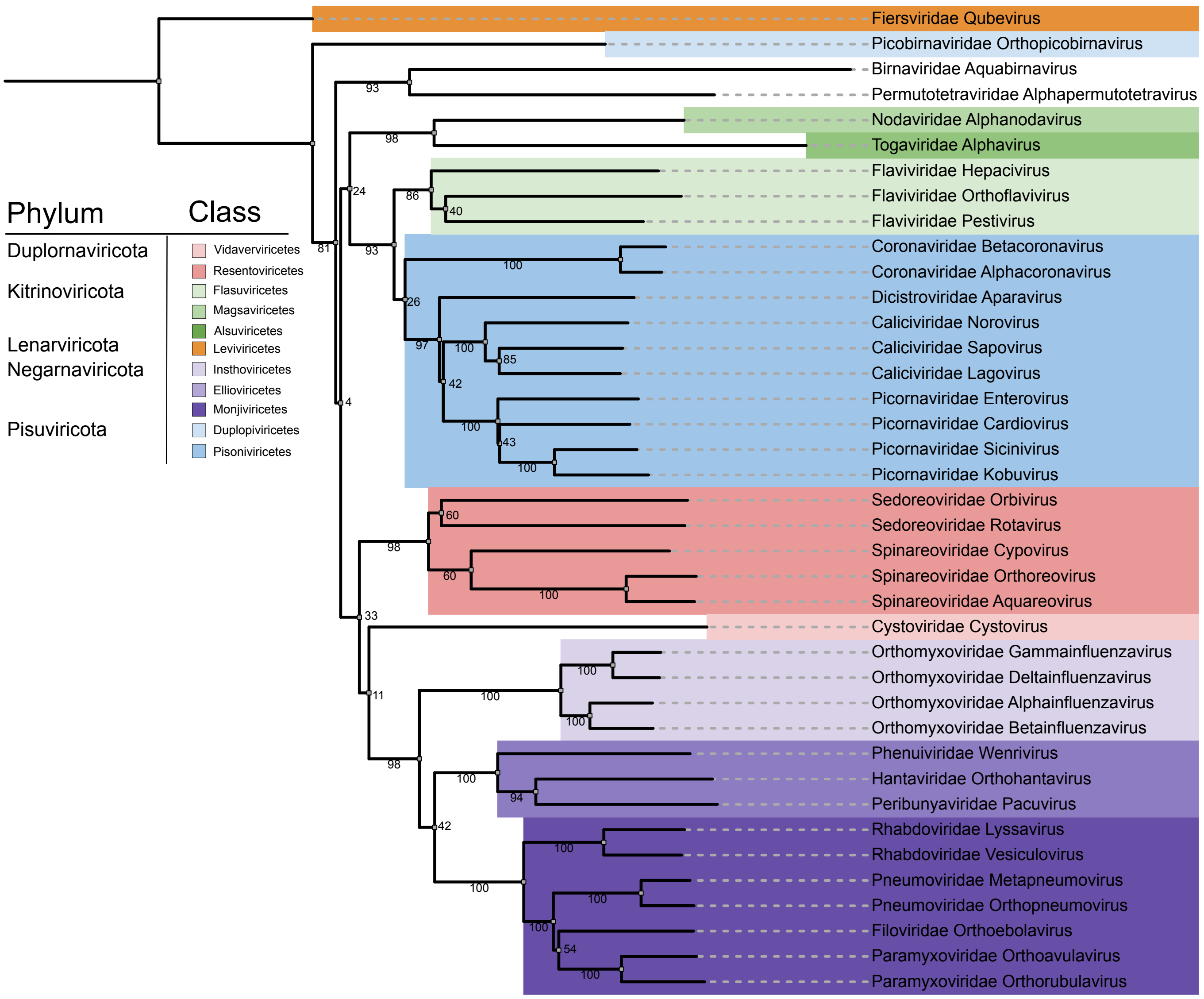

Supplementary Figure S2. Structure-based phylogenetic tree for AlphaFold3-predicted RdRP structures deduced using HSF. The branches are labelled with the viral family and genus names and coloured by the taxonomic class. The colour coding is provided on top left. The jackknifing support values are shown for branches of the tree.

Supplementary Figure S3

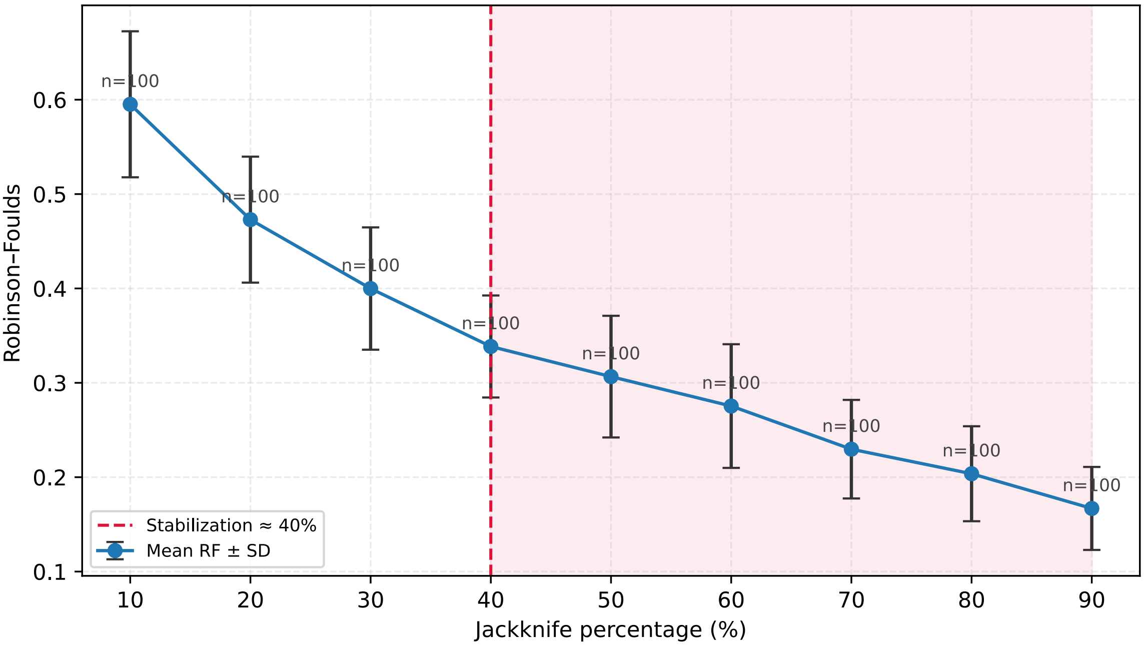

Supplementary Figure S3. Robinson–Foulds values for the jackknife sweep test. Sampling levels of 10%, 20%, 30%, 40%, 50%, 60%, 70%, 80%, and 90% of the common structural core were used. For each sampling level, the specified percentage of residues was randomly sampled 100 times, generating 100 replicate datasets. A phylogenetic tree was inferred for every replicate. Each replicate tree was then compared with the reference tree constructed from the full common structural core, and the Robinson–Foulds distance was calculated. For each sampling level, the mean Robinson–Foulds distance and its standard deviation were computed.
